# A Serendipitous Mutation Reveals the Severe Virulence Defect of a *Klebsiella pneumoniae fepB* Mutant

**DOI:** 10.1128/mSphere.00341-17

**Published:** 2017-08-23

**Authors:** Michelle Palacios, Christopher A. Broberg, Kimberly A. Walker, Virginia L. Miller

**Affiliations:** a Department of Microbiology and Immunology, University of North Carolina, Chapel Hill, North Carolina, USA; b Department of Genetics, University of North Carolina, Chapel Hill, North Carolina, USA; University of Kentucky

**Keywords:** *Klebsiella*, RamA, enterobactin, pneumonia, siderophore, yersiniabactin

## Abstract

*Klebsiella pneumoniae* is considered a significant public health threat because of the emergence of multidrug-resistant strains and the challenge associated with treating life-threatening infections. Capsule, siderophores, and adhesins have been implicated as virulence determinants of *K. pneumoniae*, yet we lack a clear understanding of how this pathogen causes disease. In a previous screen for virulence genes, we identified a potential new virulence locus and constructed a mutant (*smr*) with this locus deleted. In this study, we characterize the *smr* mutant and show that this mutation renders *K. pneumoniae* avirulent in a pneumonia model of infection. The *smr* mutant was expected to have a deletion of three genes, but subsequent genome sequencing indicated that a much larger deletion had occurred. Further analysis of the deleted region indicated that the virulence defect of the *smr* mutant could be attributed to the loss of FepB, a periplasmic protein required for import of the siderophore enterobactin. Interestingly, a Δ*fepB* mutant was more attenuated than a mutant unable to synthesize enterobactin, suggesting that additional processes are affected. As FepB is highly conserved among the members of the family *Enterobacteriaceae*, therapeutic targeting of FepB may be useful for the treatment of *Klebsiella* and other bacterial infections.

**IMPORTANCE** In addition to having a reputation as the causative agent of several types of hospital-acquired infections, *Klebsiella pneumoniae* has gained widespread attention as a pathogen with a propensity for acquiring antibiotic resistance. It is capable of causing a range of infections, including urinary tract infections, pneumonia, and sepsis. Because of the rapid emergence of carbapenem resistance among *Klebsiella* strains, there is a dire need for a better understanding of virulence mechanisms and identification of new drug targets. Here, we identify the periplasmic transporter FepB as one such potential target.

## Abstract (precis)

In addition to having a reputation as the causative agent of several types of hospital-acquired infections, *Klebsiella pneumoniae* has gained widespread attention as a pathogen with a propensity for acquiring antibiotic resistance. It is capable of causing a range of infections, including urinary tract infections, pneumonia, and sepsis. Because of the rapid emergence of carbapenem resistance among *Klebsiella* strains, there is a dire need for a better understanding of virulence mechanisms and identification of new drug targets. Here, we identify the periplasmic transporter FepB as one such potential target.

## INTRODUCTION

*Klebsiella pneumoniae* is a Gram-negative bacterium commonly classified as an opportunistic nosocomial pathogen capable of causing a variety of infections, including urinary tract infections, pneumonia, and sepsis (1–5). It is often found as a commensal resident of the gastrointestinal tract, and this is believed to be a primary source of infection (2, 6–8). Recently, *K. pneumoniae* also has been shown to be capable of causing community-acquired infections such as pyogenic liver abscesses, meningitis, and endophthalmitis (9–11). The increasing prevalence of antibiotic-resistant strains only serves to compound the clinical importance of *K. pneumoniae* and the difficulty of treating those infected with extended-spectrum β-lactamase-resistant or carbapenem-resistant strains (12–16). Resistance to carbapenems is of particular concern, as they are used as drugs of last resort to treat Gram-negative infections (12, 17).

During infection, sequestration of iron by the host limits the availability of free iron, and as a result, bacteria produce their own chelators to scavenge iron. Iron acquisition is an essential component of most bacterial pathogens, as iron is required for cellular and metabolic activities (18). Siderophores are small secreted molecules with a high affinity for ferric iron; these are classified on the basis of the chemical nature of the Fe^3+^ coordination (19). The catecholate-type siderophore enterobactin is produced by most *K. pneumoniae* strains (20, 21). However, community-acquired isolates and those that cause invasive disease typically encode additional siderophore systems (salmochelin, yersiniabactin, aerobactin) (22). Salmochelin is a C-glucosylated enterobactin produced by some isolates of *Salmonella*, *Escherichia coli*, and *Klebsiella*, and its synthesis is dependent on enterobactin. Mutants unable to produce enterobactin are also unable to produce salmochelin (23, 24). The *iroA* locus encodes enzymes necessary to modify enterobactin, as well as proteins required for salmochelin transport (25). The yersiniabactin locus is found in many invasive *K. pneumoniae* isolates and encodes a phenolate-type siderophore that was first identified as part of a pathogenicity island in *Yersinia* (26). Interestingly, in a genome-wide association study of a broad range of *K. pneumoniae* isolates, yersiniabactin was found to be the most prevalent virulence-associated locus and was found to be a predictor of infection versus carriage (22). Aerobactin is yet another siderophore produced by a smaller fraction of *K. pneumoniae* strains than either enterobactin or yersiniabactin (22). Although aerobactin has a lower affinity for Fe^3+^ than enterobactin or yersiniabactin, it is frequently produced by isolates from pyogenic liver abscesses (27).

To date, the identified virulence factors of *K. pneumoniae* primarily include capsule, lipopolysaccharide (LPS), fimbriae, and siderophores, and these factors also have been identified as virulence factors in the strain used for the studies presented here (4, 28–34). Several high-throughput studies have been done with mouse models to identify additional bacterial virulence factors (34–40). Two of these screens were signature-tagged mutagenesis (STM) screens for factors affecting gastrointestinal colonization and/or infection of the urinary tract (36, 37). These studies identified adhesins, LPS, and capsule. Another screen for gain of function when *Klebsiella* genes were expressed in *E. coli* identified a response regulator, AcrA, and LPS (40). A screen for genes expressed *in vivo* during septicemia identified genes involved in the use of siderophores (aerobactin and enterobactin) (39), and an STM screen in a model of liver abscess formation identified adhesins and regulators (38). Two of these studies focused on the identification of bacterial genes needed for survival in the lung; one approach used STM, and the other used transposon insertion site sequencing (34, 35). These screens identified capsule, LPS, siderophores, and transcriptional regulators. All of these screens also identified genes predicted to contribute generally to growth, as well as genes of unknown function.

Overall, there has been a lack of overlap in identified genes among the different screens conducted with lung, urinary tract, liver infection, and gastrointestinal colonization models. This may be due to the fact that none of the screens were saturating, or it could be indicative of mechanisms that compensate for the loss of individual genes. These findings are further complicated by the use of different infection models and different pathogen and host strain backgrounds. While typically focused on the goal of identifying previously unknown bacterial factors contributing to disease, these screens primarily identified known virulence factors of *K. pneumoniae*, as well as metabolic functions generally contributing to growth.

We previously conducted an STM screen of *K. pneumoniae* in an intranasal model of pneumonia to identify virulence genes (34). From this screen, yersiniabactin was identified as important for the abilities of our strain to colonize the lungs and to cause disseminated infection (33). In addition, a number of mutants with insertions in or near *ramA* were identified (34). RamA has been implicated in virulence and multidrug resistance in other pathogenic bacteria, and mutations in *ramA* have been associated with fluoroquinolone resistance in *K. pneumoniae* (41–44). Furthermore, a recent study reported that overexpression of RamA affects virulence and results in modified LPS (45). Thus, we sought to determine if RamA is a virulence determinant for a highly virulent *K. pneumoniae* strain. These studies found no role for *ramA* or nearby genes for virulence in a pneumonia model of infection. However, a serendipitous secondary mutation was identified, and further analysis of this mutation indicates that FepB, a periplasmic protein required for transport of enterobactin and salmochelin, is essential for virulence. Surprisingly, there were interesting differences in virulence between enterobactin synthesis mutants and the Δ*fepB* mutant.

## RESULTS

### The *smr* mutant is severely attenuated in a mouse model of pneumonia.

**FIG 1:**
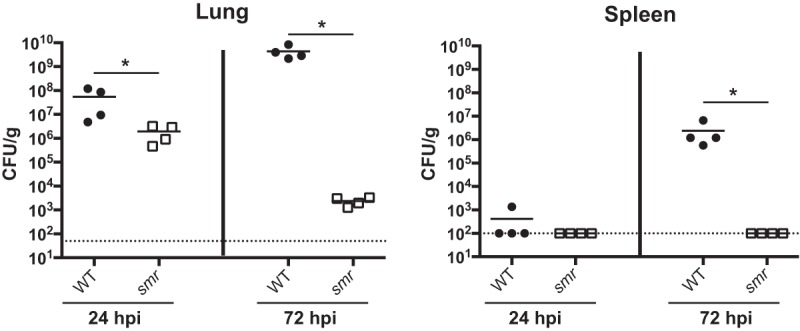
The *smr* mutant is attenuated in a mouse model of pneumonia. Mice were inoculated i.n. with 2 × 10^4^ CFU of either the WT strain (KPPR1S; black circles) or the Δ*smr* mutant (VK82; white squares). At 24 or 72 hpi, mice were euthanized and their lungs and spleens were homogenized and plated for bacterial enumeration. Each symbol represents one mouse. The dotted line indicates the limit of detection, and symbols on the dotted line indicate that CFU counts were below the limit of detection. Data are from an individual representative experiment. Mann-Whitney tests were performed for statistical analysis. *, *P* < 0.05.

A previous screen of strain KPPR1 transposon mutants identified genes required for colonization and survival in the lungs of infected mice (34). Thirteen mutants containing disruptions within *ramA* or an adjacent gene, *orf82*, failed to be recovered from the lungs and spleens of infected mice. RamA is a transcriptional regulator linked to *Salmonella* survival in RAW 264.7 macrophages and virulence in BALB/c ByJ mice (41, 42). This led us to hypothesize that the *ramA* locus is important for the ability of *K. pneumoniae* to infect the lungs. To test this, we constructed the *smr* (spontaneous multidrug resistance) mutant, where *ramA* and the two flanking genes (*orf82* and *romA*) were targeted for deletion, and tested this strain in a mouse model of pneumonia (Fig. 1). The *smr* mutant caused slightly lower bacterial burdens at 24 h postinoculation (hpi) than KPPR1 (wild type [WT]). At 72 hpi, nearly 5 logs fewer CFU were recovered from mice infected with the *smr* mutant than from WT*-*infected mice. The spleens of mice infected with the WT strain had nearly 10^7^ CFU/g of tissue, while the *smr* mutant was rarely detectable in the spleen at 72 hpi, reflecting a dissemination or systemic survival defect. Together, these data indicate that the *smr* mutant is essentially avirulent in this infection model.

### Deletions of individual genes in the targeted *smr* locus do not recapitulate the phenotype of the *smr* mutant.

**FIG 2:**
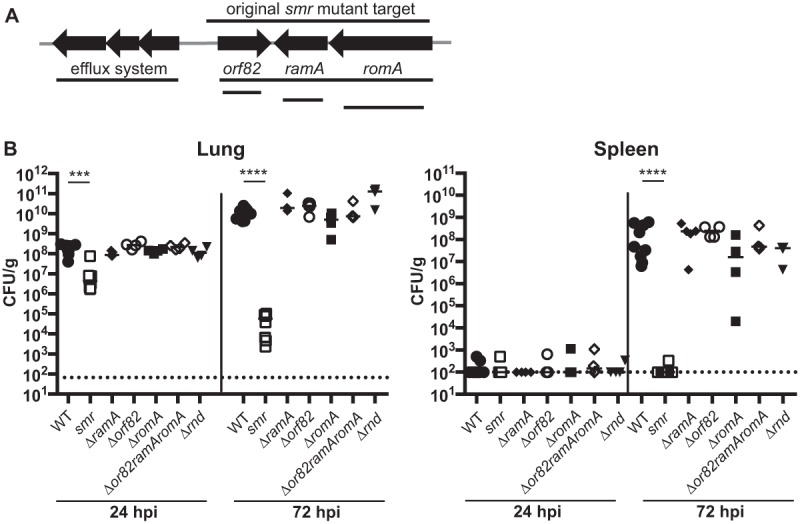
Schematic of *smr* targeted region and *in vivo* phenotypes of mutants. (A) Schematic depicting open reading frames within or adjacent to the *smr* target region (not to scale). Lines indicate the regions deleted in the mutants indicated. (B) Mice were inoculated i.n. with 2 × 10^4^ CFU of the WT strain (KPPR1S; black circles) or the Δ*smr* (VK082; white squares), Δ*ramA* (VK174; black diamonds), Δ*orf82* (VK270; white circles), Δ*romA* (VK131; black squares), Δ*orf82 ramA romA* (VK266; white diamonds), or Δ*rnd* (VK269; inverted triangles) mutant. At 24 or 72 hpi, mice were sacrificed and their lungs and spleens were homogenized and plated for bacterial enumeration. Each symbol represents one mouse. The dotted line indicates the limit of detection, and symbols on the dotted line indicate that CFU counts were below the limit of detection. These data were compiled from several independent experiments. Mann-Whitney tests were performed for statistical analysis. ***, *P* < 0.001; ****, *P* < 0.0001.

To identify the gene(s) responsible for the phenotype of the *smr* mutant, we made in-frame deletions of each of the three genes (Δ*ramA*, Δ*romA*, and Δ*orf82*) in the *smr* locus and tested them in our pneumonia model (Fig. 2A). The phenotype of all three mutant strains resembled that of the WT, suggesting that the loss of a single gene was not sufficient to affect virulence (Fig. 2B). We concluded that neither *ramA*, *orf82*, nor *romA*, individually contributed to virulence in this model or was responsible for the phenotype of the *smr* mutant.

In examining the region more closely, we noted that an RND (resistance-nodulation-division superfamily) efflux pump system was encoded just upstream of *orf82* and that the *smr* deletion could have impacted the promoter driving the expression of this locus (Fig. 2A). RND efflux systems have been shown to play roles ranging from resistance to human antimicrobial peptides in *Pseudomonas* to flagellar motility in *Burkholderia* (46). Thus, we constructed two additional mutants, one with the *rnd* genes and the other with *orf82*, *ramA*, and *romA* deleted but with the putative *rnd* promoter intact (Δ*rnd* and Δ*orf82 ramA romA*). The Δ*rnd* mutant colonized mice as efficiently as the WT strain (Fig. 2B). Intriguingly, the second mutant lacking the same three genes as the *smr* mutant (Δ*orf82 ramA romA*) also had no virulence defect.

### Sequencing of the *smr* mutant reveals a large deletion.

As targeted genetic mutations in the *smr* locus failed to recapitulate the *smr* phenotype, we hypothesized that the *smr* mutant contained a secondary mutation. Whole-genome sequencing revealed that the deletion in the *smr* mutant was larger than intended. Instead of the targeted deletion of *orf82*, *ramA*, and *romA*, a single segment of 87,290 bp spanning 78 putative open reading frames was deleted.

### A component of the enterobactin transport system contributes to virulence.

**FIG 3:**
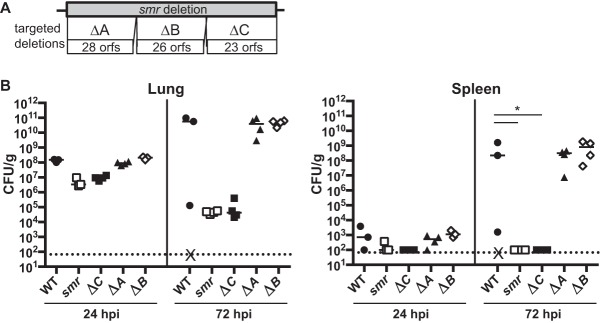
The *smr* mutant phenotype is recapitulated by a smaller targeted deletion. (A) Schematic depicting targeted subregions of the *smr* mutant (not to scale). (B) Mice were inoculated i.n. with 2 × 10^4^ CFU of the WT (KPPR1S; black circles) or the Δ*smr* (VK082; open squares), Δ*smr_A* (VK274; black triangles), Δ*smr_B* (VK275; open diamonds), or Δ*smr_C* (VK276; black squares) mutant. At 24 or 72 hpi, mice were sacrificed and their lungs and spleens were homogenized and plated for bacterial enumeration. Each symbol represents one mouse. The dotted line indicates the limit of detection, and symbols on the dotted line indicate that CFU counts were below the limit of detection. X indicates a mouse that succumbed to infection prior to 72 hpi. These data are from an individual representative experiment. Mann-Whitney tests were performed for statistical analysis. *, *P* < 0.05.

To identify the factor(s) responsible for the virulence defect of the *smr* mutant, we constructed three mutants (Δ*smr*_*A*, Δ*smr*_*B*, and Δ*smr*_*C*) each with a deletion of approximately one-third of the genes deleted in the *smr* mutant (Fig. 3A). The putative *orf* genes in each mutant are listed in Table 1. In our pneumonia model at 24 and 72 hpi, both Δ*smr*_*A* and Δ*smr*_*B* mutant-infected mice had bacterial burdens comparable to those of mice infected with the WT (Fig. 3B). However, the mice infected with Δ*smr*_*C* mutant had >1 log fewer CFU/g at 24 hpi and nearly 6 logs fewer CFU/g at 72 hpi than mice infected with the WT. Thus, the Δ*smr*_*C* mutant recapitulated the phenotype of the *smr* mutant, whereas the Δ*smr*_*A* and Δ*smr*_*B* mutants behaved like the WT strain.

**TABLE 1:** Genes deleted in breakdown mutants

Strain	Locus tag	Annotated gene product
Δ*smr_A* mutant	VK055_1987	Oxygen-insensitive NADPH nitroreductase
	VK055_1986	Hypothetical protein
	VK055_1985	Bacterial transcriptional regulator, TetR family
	VK055_1984	Metallo-beta-lactamase superfamily protein (RomA)
	VK055_1983	Bacterial regulatory helix-turn-helix, AraC family protein (RamA)
	VK055_1982	Hypothetical protein (Orf82)
	VK055_1981	Putative aldo/keto reductase
	VK055_1980	HAD^a^ ATPase, P type
	VK055_1979	Efflux transporter, RND family, MFP subunit
	VK055_1978	Efflux pump membrane transporter, BepE
	VK055_1977	Hypothetical protein
	VK055_1976	Gamma-glutamyl cysteine ligase YbdK
	VK055_1975	Hypothetical protein
	VK055_1974	Bacterial extracellular solute-binding protein
	VK055_1973	Binding-protein-dependent transport system inner membrane component
	VK055_1972	Binding-protein-dependent transport system inner membrane component
	VK055_1971	Oligopeptide/dipeptide ABC transporter, ATP binding
	VK055_1970	Oligopeptide/dipeptide ABC transporter, ATP binding
	VK055_1969	Amidase. Hydatoinase/carbamoylase family protein
	VK055_1968	EamA-like transporter family protein
	VK055_1967	Bacterial transcriptional regulator, GntR family protein
	VK055_1966	Bacterial transcriptional regulator, GntR family protein
	VK055_1965	Bacterial extracellular solute-binding
	VK055_1964	ABC transporter, permease
	VK055_1963	ABC-type amino acid transport system, permease
	VK055_1962	ABC transporter family protein
	VK055_1961	Serine 3-dehydrogenase
	VK055_1960	Aminotransferase class III family protein
		
Δ*smr_B* mutant	VK055_1959	ABC transporter family protein
	VK055_1958	ABC transporter family protein
	VK055_1957	Oligopeptide transport permease family protein
	VK055_1956	Binding protein-dependent transport system inner membrane component family protein
	VK055_1955	Bacterial extracellular solute-binding protein
	VK055_1954	Acetyltransferase family protein
	VK055_1953	Choline dehydrogenase
	VK055_1952	Betaine aldehyde dehydrogenase
	VK055_1951	Transcriptional repressor BetI
	VK055_1950	Transporter, betaine/carnitine/choline transporter family protein
	VK055_1949	*ykfE*, inhibitor of vertebrate C-type lysozyme
	VK055_1948	Bacterial regulatory helix-turn-helix, LysR family protein
	VK055_1947	Mechanosensitive ion channel family protein
	VK055_1946	Hypothetical kinase
	VK055_1945	Glycerol kinase
	VK055_1944	l-Fucose isomerase, C-terminal domain protein
	VK055_1943	Transketolase, pyrimidine binding domain protein
	VK055_1942	Thiamine pyrophosphate enzyme, C-terminal TPP^b^ binding domain protein
	VK055_1941	Hypothetical protein
	VK055_1940	Putative transcriptional regulator
	VK055_1939	Branched-chain amino acid transport system/permease component family protein
	VK055_1938	Heme ABC exporter, ATP-binding protein CcmA
	VK055_1937	Hypothetical protein
	VK055_1936	Periplasmic binding and sugar binding domain of LacI family protein
	VK055_1935	4′-Phosphopantetheinyl transferase superfamily protein, EntD
	VK055_1934	TonB-dependent siderophore receptor family protein, FepA
		
Δ*smr_C* mutant	VK055_1933	Fes
	VK055_1932	MbtH-like family protein
	VK055_1931	EntF
	VK055_1930	FepC
	VK055_1929	FepG
	VK055_1928	FepD
	VK055_1927	EntS
	VK055_1926	FepB
	VK055_1925	EntC
	VK055_1924	EntE
	VK055_1923	EntB
	VK055_1922	EntA
	VK055_1921	Proofreading thioesterase in enterobactin biosynthesis, YbdB2
	VK055_1920	Carbon starvation CstA family protein
	VK055_1919	Helix-turn-helix family protein
	VK055_1918	Hypothetical protein
	VK055_1917	Plasmid stabilization system family protein
	VK055_1916	Short-chain dehydrogenase family protein
	VK055_1915	Iron-containing alcohol dehydrogenase family protein
	VK055_1914	ABC transporter family protein
	VK055_1913	Branched-chain amino acid transport system/permease component family protein
	VK055_1912	Periplasmic binding and sugar binding domain of LacI family protein
	VK055_1911	LVIVD repeat family protein

a HAD, haloacid dehalogenase.

b TPP, thiamine pyrophosphate.

Located within the region deleted in the Δ*smr*_*C* mutant are genes necessary for the synthesis, export, and import of the siderophore enterobactin. We therefore hypothesized that a component of the enterobactin transport system was responsible for the virulence defect of the *smr* mutant. We did not believe that the siderophore itself was responsible, as an Δ*entB* mutant, which is unable to synthesize enterobactin and salmochelin, is only modestly attenuated in this mouse pneumonia model (33). The enterobactin receptor FepA also was not implicated, as FepA is encoded within the region deleted in the Δ*smr*_*B* mutant.

**FIG 4:**
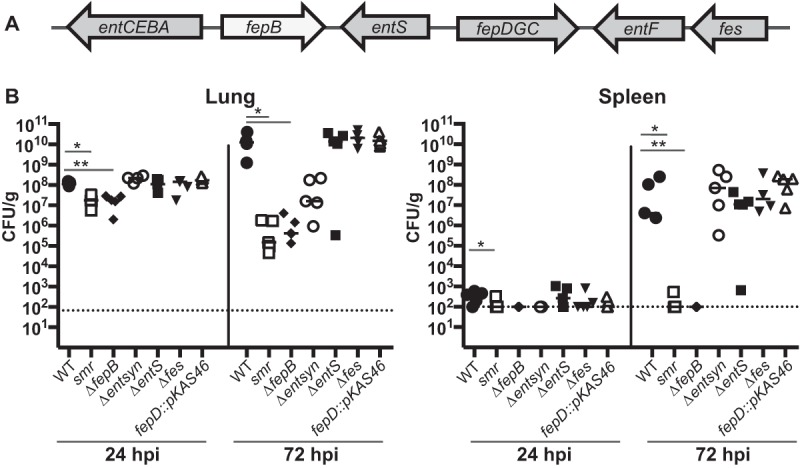
FepB is responsible for the *smr* mutant’s phenotype. (A) Schematic of the enterobactin genes located in the Δ*smr_C* region. (B) Mice were inoculated i.n. with 2 × 10^4^ CFU of the WT (KPPR1S; black circles) or the Δ*smr* (VK082; open squares), Δ*fepB* (VK412; black diamonds), Δ*ent syn* (VK321; open circles), Δ*entS* (VK411; black squares), Δ*fes* (VK320; black inverted triangles), or *fepD*::*kan* (VK413; open triangles) mutant. At 24 or 72 hpi, mice were sacrificed and their lungs and spleens were homogenized and plated for bacterial enumeration. Each symbol represents one mouse. The dotted line indicates the limit of detection, and symbols on the dotted line indicate that CFU counts were below the limit of detection. The data are from an individual representative experiment. Mann-Whitney tests were performed for statistical analysis. *, *P* < 0.05. **, *P* < 0.01.

**FIG 5:**
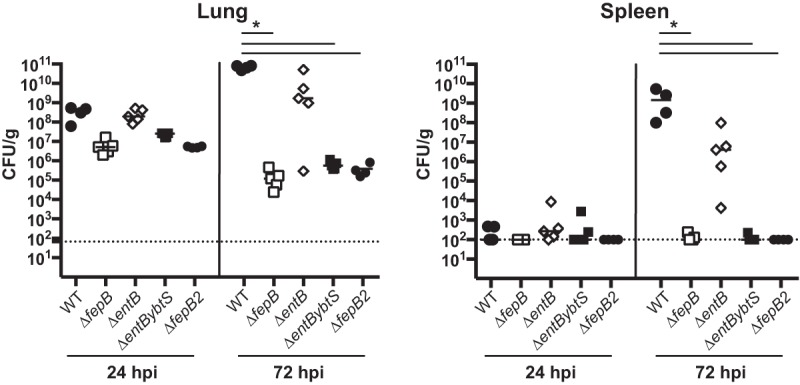
A Δ*fepB* mutant resembles a triple siderophore mutant *in vivo*. Mice were inoculated i.n. with 2 × 10^4^ CFU of the WT (KPPR1S; black circles) or the Δ*fepB* (VK412; open squares, small closed circles), Δ*entB* mutant (VK087; open diamonds), or Δ*entBybtS* (VK089; black squares) mutant. At 24 or 72 hpi, mice were sacrificed and their lungs and spleens were homogenized and plated for bacterial enumeration. Each symbol represents one mouse. The dotted line indicates the limit of detection, and symbols on the dotted line indicate that CFU counts were below the limit of detection. The data are from an individual representative experiment. Mann-Whitney tests were performed for statistical analysis. *, *P* < 0.05.

**FIG 6:**
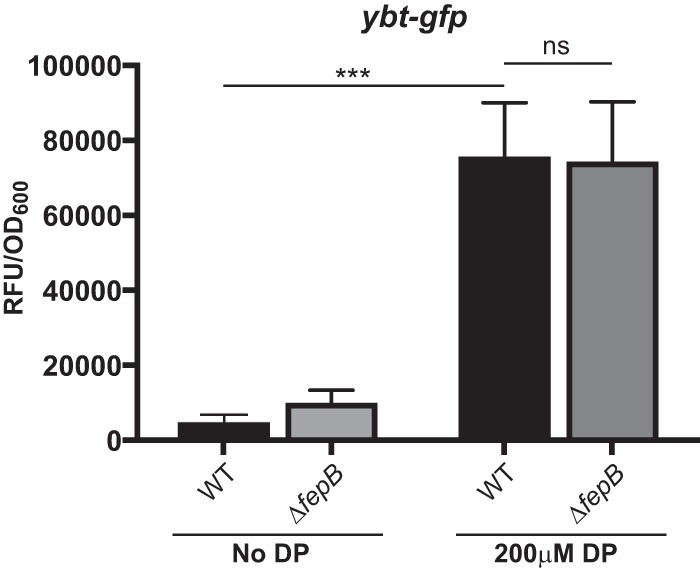
*ybtA* expression is unchanged in the Δ*fepB* mutant. The WT strain and a Δ*fepB* mutant containing the yersiniabactin synthesis gene, *ybtA*, promoter cloned into the pPROBE *gfp* reporter plasmid were grown overnight, subcultured to an OD_600_ of 0.2, and grown in LB medium for 6 h with or without 200 µM DP. These data are from strains grown in triplicate in an individual experiment. Student *t* tests were performed for statistical analysis. ***, *P* < 0.0001; ns, not significant. RFU, relative fluorescence units.

Siderophore transport involves several membrane proteins. For enterobactin, EntS and TolC are required for export, whereas FepA, FepDGC, and Fes are required for import. In addition, the periplasmic protein FepB is required for the import of both enterobactin and salmochelin. Because previous studies had implicated siderophore transport components in virulence (47), we targeted specific components of the enterobactin siderophore transport system and tested loss-of-function (Δ*entS*, Δ*fes*, Δ*fepB*, and *fepD*::pKAS46) mutants in our pneumonia model (Fig. 4A). We included a different enterobactin synthesis (Δ*ybdB2 entABEC* [referred to as Δ*entsyn*]) mutant to confirm our previous findings obtained with the Δ*entB* mutant (33). We found that only the Δ*fepB* mutant recapitulated the phenotype of the *smr* mutant, as demonstrated by the attenuation in the lungs and the lack of dissemination at 24 and 72 hpi (Fig. 4B). Consistent with previously studies, neither the Δ*entsyn* mutant (Fig. 4B) nor the Δ*entB* mutant (Fig. 5) recapitulated the *smr* phenotype (33). In addition, loss of *fepB* did not affect the expression of the yersiniabactin system (Fig. 6), consistent with results previously obtained with an enterobactin synthesis mutant (33). Thus, the periplasmic transport protein FepB contributes to virulence in a manner distinct from that of enterobactin and salmochelin uptake alone.

A variety of different approaches were used to complement the Δ*fepB* mutant, but all were unsuccessful. Plasmid-based approaches failed to complement growth under iron-depleted conditions, despite the constitutive expression of *fepB* (data not shown). We also attempted to repair the deletion, but this strain could not be obtained, for reasons we do not understand. Problems with *fepB* complementation are not unprecedented and were also reported for a *Salmonella fepB* mutant (47). To ensure that the observed phenotype of the Δ*fepB* mutant was not a consequence of secondary mutations, a second *fepB* mutant (*fepB2*) was constructed and found to recapitulate the virulence and growth phenotypes of the original *fepB* mutant (Fig. 5). Additionally, we sequenced across the deletion junction of both of the Δ*fepB* mutants and obtained the expected sequence, suggesting that a larger deletion of the region surrounding *fepB* had not occurred (data not shown). Expression of the genes adjacent to *fepB*, *entC* and *entS*, was assessed by quantitative reverse transcription-PCR. Expression of *entC* and *entS* was not detected in the Δ*fepB* mutant but was in the WT (data not shown). EntC and EntS may be needed for growth under low-iron conditions, and their lack of expression provides a possible explanation for failed complementation in *trans*. However, this alone cannot explain the attenuation *in vivo*, as a Δ*entS* mutant was not attenuated and a Δ*entC* mutant (enterobactin synthesis) had a more modest attenuation level than the Δ*fepB* mutant (Fig. 4A) (33). Thus, we conclude that deletion of *fepB* results in a phenotype distinct from that of other enterobactin system mutants.

### A *fepB* mutant resembles a Δ*entB* Δ*ybtS* double mutant.

We previously showed that a Δ*entB ΔybtS* mutant that is deficient in all siderophore production was severely attenuated (33). In comparing the defect of the Δ*fepB* mutant strain to those of other siderophore mutants, we noticed that the phenotype of the Δ*fepB* mutant was similar to that of the Δ*entB ΔybtS* mutant. Because the attenuation of the Δ*fepB* mutant was much greater than that of the Δ*entB* mutant, we hypothesized that the role of FepB is not limited to enterobactin import and that it might be involved in an additional iron acquisition system. To gain a better understanding of the relationship between the phenotypes of these mutants, we tested the Δ*fepB* mutant together with the Δ*entB ΔybtS* mutant to determine if its virulence defect resembles that of a Δ*entB ΔybtS* mutant *in vivo* and included a Δ*entB* mutant as a control (Fig. 5). The Δ*fepB* and Δ*entB ΔybtS* mutants had similar attenuation levels, which were more severe than that of the Δ*entB* mutant. This finding raises the question of whether FepB may be required for iron acquisition via systems other than enterobactin and salmochelin.

**FIG 7:**
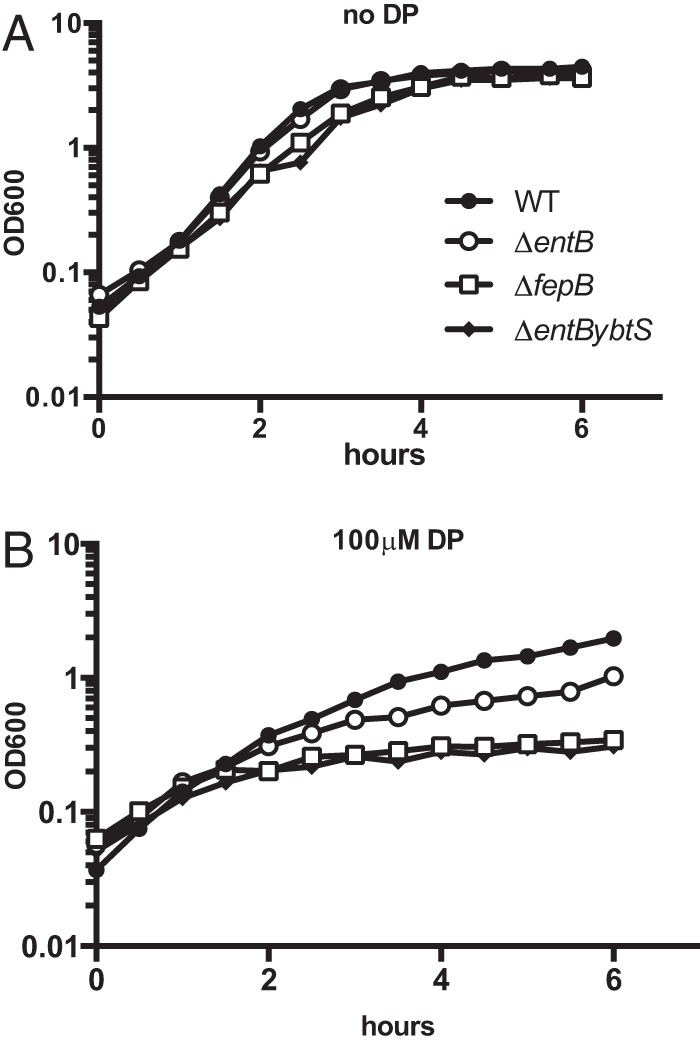
The Δ*fepB* mutant has a growth defect under iron-limited conditions. The WT strain (KPPR1S; black circles) and the Δ*fepB* (VK412; open squares), Δ*entB* (VK087; open circles), and Δ*entBybtS* (VK089; black diamonds) mutants were grown in M9-CAA (A) or in M9-CAA supplemented with 100 µM DP (B). The OD_600_ was monitored for 6 h. The data shown are from an individual representative experiment.

To address the role of FepB in iron uptake and to determine if the virulence defect could be due to reduced iron acquisition, we used an *in vitro* growth model. The Δ*fepB*, Δ*entB*, and Δ*entB ΔybtS* mutants were grown in defined medium with or without the iron-chelating agent 2,2′-dipyridyl (DP). All of the strains had similar growth rates in the absence of DP, indicating that the mutants grow normally when iron levels are sufficient (Fig. 7A). However, in the presence of DP, the growth of the Δ*fepB* and Δ*entB ΔybtS* mutants was severely restricted (Fig. 7B). Interestingly, the growth of the Δ*entB* mutant was restricted compared to that of the WT strain, but the triple siderophore (Δ*entB ΔybtS*) mutant and the Δ*fepB* mutant grew even more slowly than the Δ*entB* mutant. These data suggest that FepB contributes to growth in an iron-dependent manner that is distinct from its known role in enterobactin and salmochelin uptake.

### Yersiniabactin import is unaffected in a Δ*fepB* mutant.

**FIG 8:**
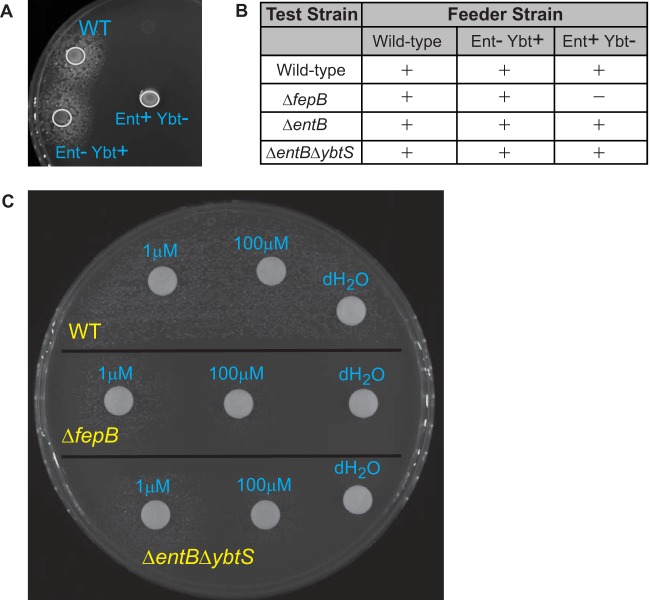
Addition of yersiniabactin restores the growth defect of the Δ*fepB* mutant under iron-limited conditions. Test strains were grown in M9-CAA and spread plated onto M9-CAA agar containing 100 µM DP. (A) Plate testing of the Δ*fepB* mutant (spread plated). Feeder (WT and Δ*entB* and Δ*ybtS* mutant) strains were then spot plated to test for complementation (growth restoration around the feeder spot). (B) Summary of results represented as + for growth and – for no growth of the WT strain, the Δ*fepB* mutant, the Δ*entB* mutant, or the Δ*entB ΔybtS* double mutant. (C) Addition of purified yersiniabactin (1 mM or 100 µM) or the dH_2_O vehicle to the WT strain, the Δ*fepB* mutant, or the Δ*entB ΔybtS* double mutant. Shown are data from an individual experiment that are representative of data obtained from several independent experiments.

The Δ*fepB* mutant had a stronger phenotype than an enterobactin/salmochelin synthesis mutant, and it resembled that of a triple siderophore mutant in both virulence and growth under iron limitation. Yersiniabactin is the only known siderophore produced by the Δ*entB* mutant but not the Δ*entB ΔybtS* mutant. Thus, we wanted to assess if the Δ*fepB* mutant is defective in yersiniabactin uptake. To do this, we performed a cross-feeding experiment to determine if the growth defect of the Δ*fepB* mutant under iron-limited conditions could be restored in the presence of yersiniabactin by coculturing the Δ*fepB* mutant with a yersiniabactin-producing strain. We predicted that if FepB is required for yersiniabactin import, a feeder strain producing yersiniabactin would be unable to restore the growth of the Δ*fepB* mutant. In this assay, test strains were spread onto M9 medium supplemented with 0.4% glucose and 0.2% Casamino acids (M9-CAA) agar containing DP and feeder strains were then spotted onto the surface of the plates. The WT and Δ*entB*, Δ*entB ΔybtS*, and Δ*fepB* mutant strains were used as test strains, and the WT and the Δ*entB* (capable of producing yersiniabactin) and Δ*ybtS* (does not produce yersiniabactin) mutants were used as feeder strains. As expected, the Δ*ybtS* mutant was not able to complement the growth defect of the Δ*fepB* mutant, as the Δ*fepB* mutant should not be able to use the enterobactin produced by this strain (Fig. 8A). The WT and the Δ*ybtS* mutant were able to complement the growth of the Δ*entB ΔybtS* mutant, as expected (Fig. 8B). Importantly, the Δ*entB* mutant and the WT were able to restore the growth of the Δ*entB* mutant (as expected), as well as the Δ*fepB* mutant. This finding suggests that yersiniabactin can still be imported by a Δ*fepB* mutant.

To determine if the complementation of the Δ*fepB* mutant’s growth defect by a yersiniabactin-producing strain in the cross-feeding experiment was due to yersiniabactin production rather than the production of other secreted bacterial products, we performed a similar experiment by spotting purified apo-yersiniabactin instead of feeder strains. As described above, test strains (WT strain and Δ*fepB* and Δ*entB ΔybtS* mutants) were spread onto M9-CAA agar containing DP. Various concentrations of apo-yersiniabactin were applied to paper discs that were placed on the agar plate to test for growth restoration and thus the ability to utilize yersiniabactin (Fig. 8C). The WT strain was able to grow even without yersiniabactin supplementation. The Δ*entB ΔybtS* and Δ*fepB* mutants did not grow around the vehicle control (distilled H_2_O [dH_2_O]) disc. However, upon the addition of yersiniabactin, the growth defect of the Δ*entB ΔybtS* mutant was restored in a concentration-dependent manner; this is an expected result because this strain is still able to import exogenous yersiniabactin. Addition of apo-yersiniabactin also restored the growth of the Δ*fepB* mutant (Fig. 8C). Together, these data suggest that FepB is not required for yersiniabactin import *in vitro* and that the virulence defect of the Δ*fepB* mutant is due to a mechanism unrelated to yersiniabactin import.

### Capsule production is not responsible for the Δ*fepB* mutant’s phenotype.

**FIG 9:**
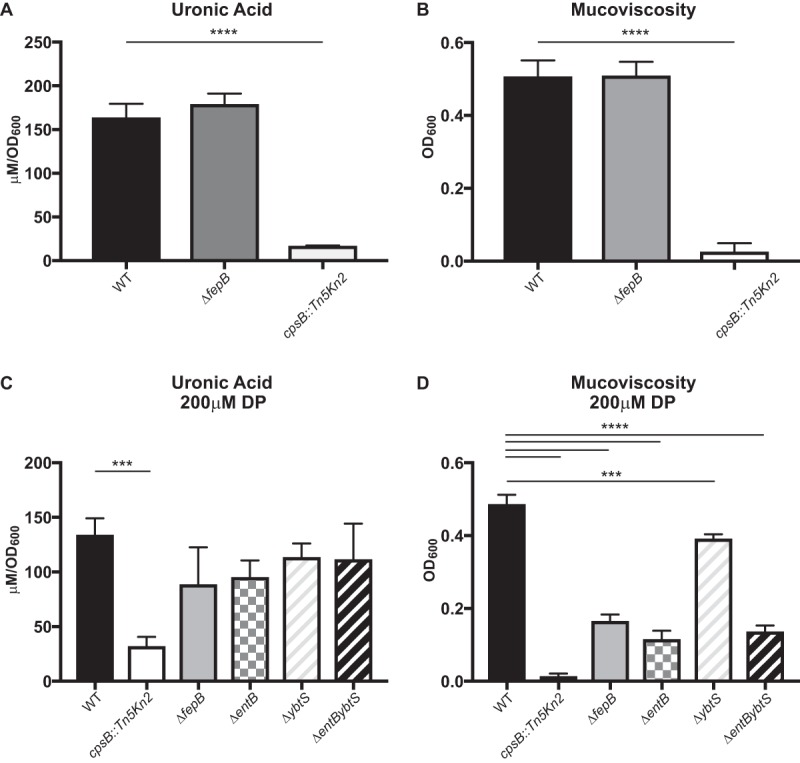
Capsule phenotype of the Δ*fepB* mutant. Overnight cultures of the WT strain, the Δ*fepB* mutant, and a capsule-deficient strain (*cpsB*::*Tn5Kn2*) were subcultured to an OD_600_ of 0.2 and grown in LB medium for 6 h, and total capsule production was measured with the uronic acid assay (A) and the low-speed centrifugation assay to measure mucoviscosity (B). These data are from strains grown in triplicate in an individual experiment. One-way analysis of variance, followed by Dunnett’s multiple-comparison test, was performed for statistical analysis. ***, *P* < 0.001; ****, *P* < 0.0001; ns, not significant.

Capsule is considered a primary virulence factor of *K. pneumoniae* (reviewed in reference 4). Therefore, to test if there was a change in capsule production that could contribute to the Δ*fepB* mutant’s phenotype, we measured its uronic acid content. When the Δ*fepB* mutant and the WT strain were grown in Luria-Bertani (LB) medium at 37°C, the same conditions used for the inoculum used in mouse experiments, there was no difference in capsule production (Fig. 9A). Similarly, when mucoviscosity was measured (another assay for capsule phenotypes), we saw no measureable difference between the WT and the Δ*fepB* mutant (Fig. 9B).

Because iron levels can affect *K. pneumoniae* capsule production (48), we decided to test if capsule production is altered in the Δ*fepB* mutant under low-iron conditions. All four siderophore system (Δ*fepB*, Δ*entB*, Δ*ybtS*, and Δ*entB ΔybtS*) mutants had a modest, nonsignificant reduction in capsule production (Fig. 9C). The mucoviscosity of the siderophore system mutants was also lower than that of the WT (Fig. 9D). Importantly, there was no difference between the capsule production levels of the Δ*fepB* and Δ*entB* mutants. How FepB affects virulence is not clear, but it does not appear to be related to the amount of capsule produced (Fig. 9A and C)) or the mucoviscosity of the capsule (Fig. 9B and D), as the uronic acid content and sedimentation of the Δ*fepB* mutant were comparable to those of the enterobactin synthesis mutant, which is only modestly attenuated.

## DISCUSSION

The repertoire of confirmed *K. pneumoniae* virulence factors has changed little during the past 2 decades (2, 4). Although a number of large screens for *K. pneumoniae* virulence determinants have been performed (34–40), unfortunately, there have been few follow-up analyses of the results of these screens. In a screen of signature-tagged mutants in a pneumonia model of infection, we identified a locus that included *ramA* as potentially important for virulence (34), and a recent study suggested that overexpression of *ramA* affects virulence and leads to LPS modifications (45). In this study, we constructed a mutant (*smr*) with this locus deleted and found that it was cleared from the lungs following intranasal inoculation and that it was unable to spread systemically. Why deletion of *ramA* or the surrounding genes did not result in a virulence defect in the lungs and/or spleen when 11 insertions in this region were identified in the STM screen remains a mystery (34). One possibility is that in the STM screen, each insertion mutant was screened essentially in competition with 95 other mutants, most of which behave like the WT strain. Therefore, a *ramA* mutant may have a competitive disadvantage when at a ratio of ~1:100 with the WT but will not exhibit a defect when inoculated on its own. RamA has been implicated in the regulation of pathways important for multidrug resistance (43, 44), and thus, it may still be important in the context of antibiotic treatment or in a strain background that is not hypervirulent.

Subsequent analysis of the *smr* mutant indicated that the virulence defect was due not to deletion of the *ramA* locus but rather to the deletion of *fepB*, a gene encoding a protein required for enterobactin and salmochelin import (49–51). The *fepB* mutant had a more severe growth defect in iron-limited medium and a more severe *in vivo* defect than an enterobactin synthesis (Δ*entB*) mutant; the Δ*entB* mutant would also be deficient in salmochelin production. The contributions of the siderophores enterobactin, salmochelin, and yersiniabactin to *Klebsiella* virulence have been examined previously, and individually, they were found to contribute only minimally to infection (32, 33, 52). The data presented here reveal that while enterobactin/salmochelin may be dispensable for the virulence of a strain also able to produce yersiniabactin in a *K. pneumoniae* lung infection model, the enterobactin/salmochelin importer FepB is necessary to establish infection. Furthermore, under both *in vitro* and *in vivo* conditions, the Δ*fepB* mutant resembles a Δ*entB ΔybtS* mutant, which is unable to produce any of the three siderophores encoded by this strain (enterobactin, salmochelin, and yersiniabactin). Together, these observations suggest that FepB contributes to virulence and growth under iron limitation in an unanticipated way.

Siderophores are synthesized in the cytoplasm and require machinery for export and subsequent import following iron sequestration. Enterobactin is synthesized by EntABCDEF and is exported to the periplasm via the inner membrane protein EntS and subsequently through the outer membrane via the membrane channel protein TolC (53). Once bound to ferric iron, enterobactin (enterobactin-Fe^3+^) binds the outer membrane siderophore receptor FepA and is translocated into the periplasm by a TonB-dependent mechanism. In the periplasm, enterobactin-Fe^3+^ then binds the periplasmic chaperone FepB and is shuttled to the inner membrane, where it interacts with the inner membrane transport complex FepDGC and is ultimately released into the cytoplasm (50, 54, 55). Salmochelin utilizes a similar export apparatus but is imported via the bacterial outer membrane receptor IroN, and then FepB shuttles it to FepDGC (56). Export and yersiniabactin import appear to be similar, although several steps in yersiniabactin transport remain to be elucidated (53). Specifically, no periplasmic protein (FepB equivalent) has been identified in the yersiniabactin import system. Because of the similarities in the phenotypes of the Δ*fepB* and triple siderophore mutants and because no FepB equivalent has been identified in the yersiniabactin import system, we initially hypothesized that FepB may be involved in yersiniabactin import. However, our results show that a Δ*fepB* mutant can still utilize yersiniabactin for growth *in vitro*, and thus, the role of FepB in growth under iron limitation and virulence remains unclear. A recent crystal structure of FepB indicates that it can form a trimer (57) and thus possibly could coordinate a target other than enterobactin-Fe^3+^, but this has yet to be demonstrated.

A contribution of the periplasmic enterobactin transporter FepB to pathogenesis also was observed in *Salmonella enterica* (47). *Salmonella* produces both enterobactin and salmochelin, and both siderophores require FepB for import (25). However, Nagy et al. found that a *fepB* mutant had lower colonization levels in mice than a *fepA-iroN* double mutant (encoding the outer membrane receptors for enterobactin and salmochelin) in a gastric model of infection (47, 58). This is comparable to our results obtained with *K. pneumoniae* and suggests that the role of FepB in virulence extends beyond siderophore transport. The fact that this phenomenon has been reported in two Gram-negative pathogens hints that this may be a conserved mechanism in other bacterial species. One possible explanation for this observation is that in a Δ*fepB* mutant, enterobactin is not recycled properly and accumulates extracellularly and perhaps this is detrimental to the bacteria, given that enterobactin can enhance copper toxicity (59). However, in this scenario, the Δ*smr_C* mutant (which is a Δ*entB* Δ*fepB* double mutant and has other genes [listed in Table 1] deleted) should not have this phenotype, as it would be unable to produce enterobactin. However, the data presented here suggest that this is not the case, as the Δ*smr_C* mutant has a virulence defect comparable to that of a Δ*fepB* mutant.

Interestingly, recent studies have noted that the complement of siderophore systems produced by an individual strain of *K. pneumoniae* has a significant impact on its ability to colonize versus its ability to cause an infection or its ability to cause invasive disease associated with the hypervirulence phenotype (22). In an analysis of a broad sampling of over 300 strains, only 33% of an individual strain’s genome is part of the core *Klebsiella* genome, and the remaining 67% is composed of “accessory” genes that vary significantly from strain to strain (22). Until recently, the gene profiles necessary to cause the different types of infections associated with *K. pneumoniae* were not clear. However, recent bioinformatics analyses of large strain collections, combined with information on the type of infection, have revealed that some specific gene profiles are associated with colonization versus infection versus invasive disease. For example, the presence of *rmpA* (a regulator of capsule), as well as the genes required for the production and use of the siderophores aerobactin, salmochelin, and yersiniabactin, was highly associated with strains isolated from infections versus carriage alone (22). Interestingly, an additional five loci were associated with invasive infections (versus noninvasive infections or carriage), including *fepB*. This is consistent with the requirement we observed for *fepB* to cause disseminated infection in mice and what has been observed in *Salmonella* (47).

With antibiotic resistance on the rise, the development of new therapeutics to combat infection by multidrug-resistant bacteria is an urgent need (60). Siderophore systems present an attractive target for drug development because of the conservation of these systems among Gram-negative pathogens (61). Immunization with the yersiniabactin receptor FyuA or the siderophores themselves (yersiniabactin and aerobactin) was protective when tested in a murine model of *E. coli* urinary tract infection (62–64). FepB may be an especially attractive target to consider for drug development, as it is required for disseminated infections and is found in a wide variety of bacteria. In addition to being potential targets for drug development, siderophores represent an attractive system to exploit as a drug delivery mechanism to overcome the permeability barrier of the outer membrane. In essence, the siderophore can be used as a “Trojan horse” to target a siderophore-drug conjugate to the siderophore-iron transport systems (61). This would allow the delivery of drugs to the periplasm and potentially to the cytoplasm. From the work presented here and with *Salmonella*, one such periplasmic target could be FepB itself. Drug-siderophore conjugates have been developed, and a catechol-cephalosporin conjugate, cefiderocol (S-649266), was found to have lower MIC_90_s than the antibiotics cefepime, piperacillin-tazobactam, and meropenem when tested against several Gram-negative bacteria, including multidrug- and carbapenem-resistant strains (65–67). Cefiderocol displayed antibacterial properties when tested *in vivo* and is currently being tested in a phase 3 clinical trial against carbapenem-resistant Gram-negative infections in humans (66, 68). Thus, investigations probing the mechanisms of siderophore transport can provide the basis for promising new therapeutics.

## MATERIALS AND METHODS

### Ethics statement.

Mouse experiments were conducted in accordance with the *Guide for the Care and Use of Laboratory Animals* of the National Institutes of Health (69). All animal studies were approved by the Institutional Animal Care and Use Committee at the University of North Carolina (UNC) at Chapel Hill (protocols 11-127 and 14-110). All efforts were made to minimize suffering. Animals were monitored daily following inoculation and were euthanized upon exhibiting signs of morbidity.

### Bacterial strains and culture conditions.

The bacterial strains and plasmids used in this study are described in Table 2. The WT parental strains are KPPR1, a Rif^r^ derivative of ATCC 43816 (34), and KPPR1S, a Str^r^ derivative of KPPR1; they have identical growth characteristics *in vitro* and *in vivo*. *K. pneumoniae* strains were grown aerobically in LB medium or M9-CAA overnight at 37°C. Where indicated, 100 or 200 µM DP (Sigma-Aldrich, St. Louis, MO) was added to M9 or LB medium, respectively, to deplete the available iron. Antibiotics were added to the medium as appropriate at the following concentrations: kanamycin, 50 µg/ml (Kan_50_); rifampin, 30 µg/ml (Rif_30_); streptomycin, 500 µg/ml (Strep_500_). Bacterial growth was monitored by measuring the optical density at 600 nm (OD_600_).

**TABLE 2:** Bacterial strains and plasmids used in this work

Strain or plasmid	Description	Reference
*E. coli*		
DH5α	F^−^ ϕ80d*lacZ*ΔM15 Δ(*lacZYA-argF*)*U169 deoP recA1 endA1 hsdR17*(r_K_^−^ m_K_^−^)	Invitrogen
S17-1 λ*pir*	Tp^r^ Str^r^ *recA thi pro hsdR hsdM*^*+*^ RP4-2-Tc::Mu Km Tn*7* λ*pir* (lysogen)	72
		
*K. pneumoniae*		
KPPR1	Rif^r^ derivative of ATCC 43816	34
KPPR1S	Str^r^ derivative of KPPR1	This work
VK060	KPPR1 *cpsB*::Tn*5Kn2*	34
VK082	*smr* mutant	This work
VK087	KPPR1 Δ*entB*	33
VK088	KPPR1 Δ*ybtS*	33
VK089	KPPR1 Δ*entB ΔybtS*	33
VK131	KPPR1 Δ*romA*	This work
VK174	KPPR1S Δ*ramA*	This work
VK266	KPPR1S Δ*orf82* Δ*ramA* Δ*romA*	This work
VK269	KPPR1S Δ*rnd*	This work
VK270	KPPR1S Δ*orf82*	This work
VK274	KPPR1S Δ*smr_*A	This work
VK275	KPPR1S Δ*smr_*B	This work
VK276	KPPR1S Δ*smr_*C	This work
VK320	KPPR1S Δ*fes*	This work
VK321	KPPR1S Δ*ybdB2 entABEC* (Δ*entsyn*)	This work
VK411	KPPR1S Δ*entS*	This work
VK412	KPPR1S Δ*fepB*	This work
VK413	KPPR1S *fepD*::pKAS46	This work
VK555	KPPR1S Δ*fepB2*	This work
		
Plasmids		
pKAS46 vector	Kanamycin resistance, suicide vector, *rpsL*^+^	70
pK03 vector	*sacB*, temperature-sensitive origin of replication	73
pPROBE vector	Km^r^, *gfp* expression vector	77
pKO3Δ*romA*	*romA* flanking region in pKO3	This work
pKO3Δ*ram*KO	*smr* flanking region in pKO3	This work
pKAS46Δ*ramA*	*ramA* flanking region in pKAS46	This work
pKAS46Δ*orf82*	*orf82* flanking region in pKAS46	This work
pKAS46Δ*orf82ramAromA*	*orf82 ramA romA* flanking region in pKAS46	This work
pKAS46Δ*rnd*	*rnd* flanking region in pKAS46	This work
pKAS46Δ*fepB*	*fepB* flanking region in pKAS46	This work
pKAS46Δ*smr_*A	*smr_A* flanking region in pKAS46	This work
pKAS46Δ*smr_*B	*smr_B* flanking region in pKAS46	This work
pKAS46Δ*smr_*C	*smr_C* flanking region in pKAS46	This work
pKAS46Δ*fes*	*fes* flanking region in pKAS46	This work
pKAS46Δ*entS*	*entS* flanking region in pKAS46	This work
pKAS46Δ*ybdB2entABEC*	*ybdB2 entABEC* (Δ*entsyn*) flanking region in pKAS46	This work
p*fepD*::pKAS46	Disruption of *fepD*	This work
pY4	*ybtA* promoter in pPROBE	33

### Construction of bacterial mutants.

Mutations in KPPR1S (Δ*ramA* Δ*orf82*, Δ*orf82*Δ*ramA*Δ*romA*, Δ*entS*, Δ*ybdB2 entABEC* [referred to as Δ*entsyn*], Δ*fes*, Δ*fepB*, Δ*smr*_*A* Δ*smr*_*B*, and Δ*smr*_*C*) were generated by allelic exchange by using pKAS46, a suicide vector that allows the use of streptomycin for counterselection (70, 71). Sequences up- and downstream (~500 bp each) were generated by PCR with the primer sets indicated in Table 3, cloned into pKAS46, and confirmed by sequence analysis. Overnight cultures of KPPR1S and *E. coli* S17-1 λ*pir* (72) carrying a derivative of pKAS46 were mixed, collected by centrifugation, plated on LB agar (no antibiotics), and grown overnight at 37°C. Transconjugants were selected by plating on LB agar with Rif_30_ and Kan_50_. Several Rif^r^ Kan^r^ colonies were grown for 5 to 6 h in LB medium (no antibiotics) and then plated on LB agar with Strep_500_ to select for transconjugants that had excised the plasmid. Kan^s^ clones were screened by PCR to verify the loss of the targeted gene(s).

**TABLE 3:** Primers used in this study

Primer	Sequence^a^ (5′ to 3′)	Description
MP66	TGACTA**GATATC**GCTGATTACCGAAGCGGACTG	5′ flank forward Δ*ramA*
MP67	TGCATA**TCTAGA**GGAAATCGTCATATGCTCTCT	5′ flank reverse Δ*ramA*
MP68	TGCATA**TCTAGA**CACTGAGGCGCGCCTCTCCTG	3′ flank forward Δ*ramA*
MP69	TCGATA**GCGGCCGC**CGACTGGCGCTGTACATCGCG	3′ flank reverse Δ*ramA*
MP114	TGACTA**GATATC**TCGCCCGAGGGCGTCGTAAAC	5′ flank forward Δ*orf82*
MP71	TGCATA**TCTAGA**CTCGAGCGGTAAACCAGGAGA	5′ flank reverse Δ*orf82*
MP72	TGCATA**TCTAGA**CAGTGGATGTTTCATGTCATG	3′ flank forward Δ*orf82*
MP115	TCGATA**GCGGCCGC**GGGATGAACCGTATCAACGGC	3′ flank reverse Δ*orf82*
MP124	TGACTA**GATATC**CGATTTTGCCTGCTATGCGCA	5′ flank forward Δ*rnd*
MP125	TGCATA**TCTAGA**CATCGGCGGGGGTAAGCGCGG	5′ flank reverse Δ*rnd*
MP126	TGCATA**TCTAGA**GTTCACCCGGTCGCCCAGCGG	3′ flank forward Δ*rnd*
MP127	TCGATA**GCGGCCGC**GCCACGGCAGGTCTGGCAGCA	3′ flank reverse Δ*rnd*
MP103	TGACTA**GATATC**GGCGTCGTAAACTTTGGGTTA	5′ for Δ*orf82 ramA romA*
MP104	TGCATA**TCTAGA**TTCCAGTGGATGTTTCATGTC	5′ rev Δ*orf82 ramA romA*
MP105	TGCATA**TCTAGA**CTGACCAGACAAAAGCCCCCA	3′ for Δ*orf82 ramA romA*
MP106	TCGATA**GCGGCCGC**CGACAGCTGGCACATTTCGTT	3′ rev Δ*orf82 ramA romA*
MP171	TCGATA**GCGGCCGC**CTGTGCGCTCCCTGCGCCATG	5′ flank forward *smr*ΔA
MP172	TGCATA**TCTAGA**CTGGCGAAGTAGGGGAGGGGG	5′ flank reverse *smr*Δ*A*
MP173	TGCATA**TCTAGA**ACCGAGATTTAATCTCTCCAC	3′ flank forward *smr*ΔA
MP174	TGACTA**GATATC**TCCAACTTTTGGGGTGCAGTC	3′ flank reverse *smr*Δ*A*
MP175	TGACTA**GATATC**CCATGCGCTTGCGCGGGCCTA	5′ flank forward *smr*ΔB
MP176	TGCATA**TCTAGA**GCTTACGATATTTCCAATCCG	5′ flank reverse *smr*Δ*B*
MP177	TGCATA**TCTAGA**TGCGCCTCATTAAGCGGGTCC	3′ flank forward *smr*ΔB
MP178	TCGATA**GCGGCCGC**AATGACAGAATGTTAAGGACA	3′ flank reverse *smr*Δ*B*
MP179	TGACTA**GATATC**TGCGCCTCATTAAGCGGGTCC	5′ flank forward *smr*ΔC
MP180	TGCATA**TCTAGA**AGTCACGCTATACATAGGGTT	5′ flank reverse *smr*Δ*C*
MP181	TGCATA**TCTAGA**GCGCACCCTGGCGGAGCCACT	3′ flank forward *smr*Δ*C*
MP182	TCGATA**GCGGCCGC**ATTAACGACAGGTTGCGCGAA	3′ flank reverse *smr*Δ*C*
MP282	TGACTA**GATATC**AGAATTTAACAACACCGAAAC	5′ flank forward Δ*ybdB2 entABEC*
MP192	TGCATA**TCTAGA**ACCGCGGTGCTGGGCTAAGAA	5′ flank reverse Δ*ybdB2 entABEC*
MP193	TGCATA**TCTAGA**AGCCAGTGACGTTTCCATATC	3′ flank forward Δ*ybdB2 entABEC*
MP194	TCGATA**GCGGCCGC**GCAACCTCGCTCCACTGGCGC	3′ flank reverse Δ*ybdB2 entABEC*
MP195	TGACTA**GATATC**GGATATAGAGCTCGGAAGGCT	5′ flank forward Δ*fepB*
MP196	TGCATA**TCTAGA**GAAGTTCACGTCATCGCATCC	5′ flank reverse Δ*fepB*
MP197	TGCATA**TCTAGA**CTGTTCGGCTAACGCGGGCTG	3′ flank forward Δ*fepB*
MP198	TCGATA**GCGGCCGC**CGCTGGCGCTTGTCGGCGTGC	3′ flank reverse Δ*fepB*
MP199	TGACTA**GATATC**GCGCTCTGCTGGTGCTCCAGC	5′ flank forward Δ*entS*
MP200	TGCATA**TCTAGA**ATTGTCAACGAAAGTTAAGTA	5′ flank reverse Δ*entS*
MP201	TGCATA**TCTAGA**GGATTGTCGGTTCATTACAGC	3′ flank forward Δ*entS*
MP202	TCGATA**GCGGCCGC**CGGGTGAGCGTCTGCATCAGC	3′ flank reverse Δ*entS*
MP207	TGACTA**GATATC**GCGCGGCAACCAGCGGTAAAC	5′ flank forward Δ*fes*
MP208	TGCATA**TCTAGA**GGCCAACGCGAACCGATTATT	5′ flank reverse Δ*fes*
MP244	TGCATA**TCTAGA**TGCGCCTCATTAAGCGGGTCC	3′ flank forward Δ*fes*
MP231	TCGATA**GCGGCCGC**AATGACAGAATGTTAAGGACA	3′ flank reverse Δ*fes*
MP313	TGACTA**GATATC**CCTTAGCCGCCGCGCTTA	5′ forward *fepD*::*kan*
MP314	TGCATA**TCTAGA**TTGCGGGTGAGCGTCTGC	3′ reverse *fepD*::*kan*
ramKOA5′INsmaI	TCC**CCCGGG**ACCGCTTTGACGGTCAT	5′ flank forward *smr*
ramKOA3′IN2	CGCGGTAGATTCCAAACATA	5′ flank reverse *smr*
ramKOB5′IN	ATCCTGACCAGACAAAAGCCCCATCC	3′ flank forward *smr*
ramKOB3′INSma	TCC**CCCGGG**GACAGCTGGCACATTTC	3′ flank reverse *smr*
romA5′inXba	GC**TCTAGA**GCCAGTCCGCTTCGGTAA	5′ flank forward Δ*romA*
romA5′in	CGACTTTCATCGCTTTCCTAATA	5′ flank reverse Δ*romA*
romA3′in	CGTCATATGCTCTCTCCTCTGAT	3′ flank forward Δ*romA*
romA3′inXbaI	GC**TCTAGA**GCACAGCTTAGCCAGGTG	3′ flank reverse Δ*romA*

a Restriction sites are in bold.

An insertional disruption of the *fepDCG* operon was constructed in KPPR1S (*fepD*::pKAS46) by plasmid integration into the *fepD* gene. A DNA fragment generated by PCR with primers MP313 and MP314 (Table 3) was cloned into pKAS46. The resulting plasmid, pKAS46*fepD*::*kan*, was conjugated into KPPR1S as described above. Colonies with integration of the plasmid on the chromosome were identified by plating on LB agar with Rif_30_ and Kan_50_.

Isogenic mutants of KPPR1 (Δ*romA* and Δ*smr*) were generated by allelic exchange with pKO3 as previously described (73). pKO3 is a vector that allows the use of sucrose as a positive selection for the loss of the vector. DNA fragments were amplified by PCR with the primer sets indicated in Table 3 and cloned into pKO3, generating plasmids pKO3Δ*romA* and pKO3Δ*smr*.

### Whole-genome sequencing of the *smr* mutant.

Total DNA from the *smr* mutant (VK82) was isolated with a genomic DNA purification kit (Qiagen), and the sample was submitted to the UNC High-Throughput Sequencing Facility for sequencing. An Illumina HiSeq 2000 instrument generated 2 × 75-bp paired-end reads. A mapped genome assembly was produced with the “Map Reads to Reference” tool in CLC Genomics Workbench v7.5.1 by using the published KPPR1 genome as the template (74). The *smr* mutant and KPPR1 parent strain genomes were then compared with the “Basic Variant Detection” tool in CLC Genomics Workbench to identify mutations in the *smr* strain. Mutations were visualized by aligning these genomes with Mauve (75).

### Murine model of pneumonia.

Five- to 8-week-old, female C57BL/6 mice (Jackson Laboratories) were anesthetized by intraperitoneal injection with 200 µl of a mixture of ketamine (6.66 mg/kg) and xylazine (10.6 mg/kg). Overnight bacterial cultures were diluted in phosphate-buffered saline (PBS), and 20 µl was inoculated intranasally (i.n.) in two 10-µl aliquots for a total of ~2 × 10^4^ CFU/mouse as previously described (34). At 24, 48, or 72 hpi, mice were euthanized by a lethal injection of 200 µl of sodium pentobarbital (150 mg/kg). Organs were removed, homogenized in PBS, serially diluted, and plated to quantify the number of CFU/g of tissue.

### Mucoviscosity assay.

Mucoviscosity was determined as previously described (35, 76). Briefly, overnight cultures were grown in LB medium, subcultured to an OD_600_ of 0.2 in fresh medium, and grown at 37°C. After 6 h, cultures were normalized to 1.0 U of OD/ml and centrifuged for 5 min at 1,000 × *g* and the OD_600_ of the supernatant was measured.

### Extraction and quantification of capsule.

Uronic acid was extracted and quantified as previously described (28). Briefly, overnight cultures were grown in LB medium, subcultured to an OD_600_ of 0.2 in fresh medium, and grown at 37°C. After 6 h, 500 µl of culture was added to 100 µl of 1% Zwittergent–100 mM citric acid and incubated at 50°C for 20 min. Cells were pelleted, and 300 µl of the supernatant was added to 1.2 ml of absolute ethanol, incubated at 4°C for 20 min, and centrifuged for 5 min at maximum speed. The pellet was resuspended in 200 µl of dH_2_O, added to 1.2 ml of 12.5 mM sodium tetraborate in sulfuric acid, and incubated for 5 min at 100°C. A 20-µl volume of 0.15% 3-phenylphenol was added, and the absorbance at 520 nm was measured. The glucuronic acid content was determined from a standard curve of glucuronic acid (Sigma-Aldrich, St. Louis, MO) and expressed in micromoles per OD unit.

### Measurement of promoter activity.

Expression of the yersiniabactin-encoding locus was assessed *in vitro* with a transcriptional *gfp* reporter containing the sequence 500 bp upstream of the *ybtA* promoter cloned into pPROBE (33, 77). The bacteria were grown overnight at 37°C in LB medium, subcultured to an OD_600_ of 0.2, and grown for 6 h with or without 200 μM DP. All strains were assayed in triplicate. Fluorescence was detected with a Synergy HT microplate reader (BioTek Instruments, Winooski, VT) and measured in relative fluorescence units per OD_600_ unit.

### *In vitro* growth curves.

To monitor bacterial growth, bacterial strains were grown overnight in M9-CAA at 37°C, subcultured to an OD_600_ of 0.05 in fresh medium in 250-ml flasks, and grown with aeration for 6 h at 37°C. OD_600_ readings were recorded at the intervals indicated. Medium was supplemented with 100 μM DP to examine bacterial growth under iron-limiting conditions.

### Cross-feeding assay.

To determine if secreted siderophores could restore the growth of siderophore mutants in iron-depleted medium, a cross-feeding assay was performed as previously described, with minor modifications (78). Bacteria were grown overnight at 37°C in M9-CAA. Approximately 1 × 10^5^ CFU of each test strain was spread onto M9-CAA agar plates containing 100 μM DP. Feeder strains were then spotted (2.5 µl of overnight culture) onto the agar, and the plates were incubated at 37°C overnight.

To determine if purified yersiniabactin could restore the growth of siderophore mutants in iron-depleted medium, test strains were spread on M9-CAA agar as described above. Iron-free yersiniabactin (EMC Microcollections, Germany) was resuspended in ethanol, and 10 µl of either 1 mM or 100 µM yersiniabactin (diluted in dH_2_O) was spotted onto filter disks on the plate to assess yersiniabactin-dependent growth complementation.

### Statistical analysis.

Statistical analyses were performed with GraphPad Prism, version 6.0 (GraphPad, San Diego, CA).
